# Supplementary figures and images for: Daxx mediated histone H3.3 deposition on HSV-1 DNA restricts genome decompaction and the progression of immediate-early transcription
Source: PLoS Pathog. 2025 Aug 20;21(8):e1012501. doi: 10.1371/journal.ppat.1012501 (PMC12393724; doi:10.1371/journal.ppat.1012501)

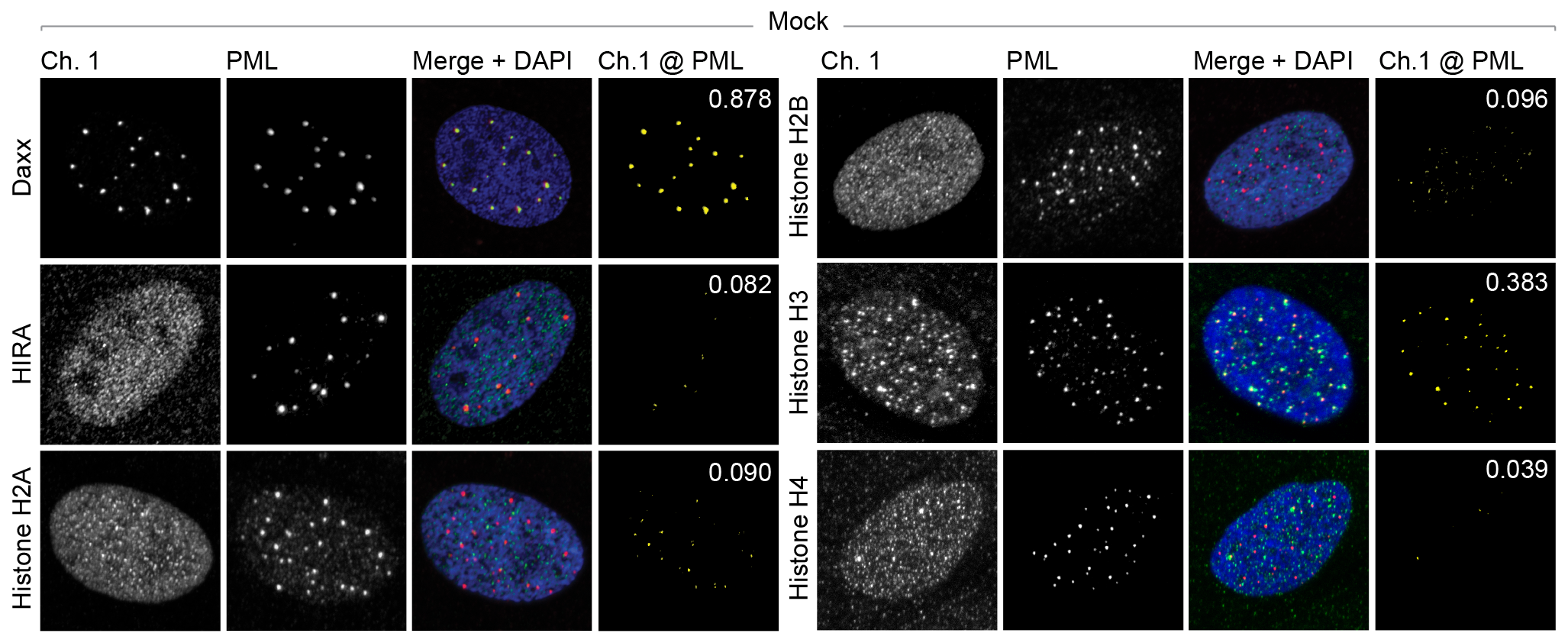

Supplement: S1 Fig — Confocal microscopy images of data presented in Fig 1B. Mock-treated HFt cells were stained for Daxx, HIRA, histones H2A, H2B, H3, or H4 (Channel 1 [Ch.1]; green, as indicated) and PML (red) by indirect immunofluorescence. Nuclei were stained with DAPI (blue). Cut mask (yellow) highlights regions of colocalization between cellular proteins of interest and PML; weighted colocalization coefficient shown. (TIF) [file ppat.1012501.s001.tif]

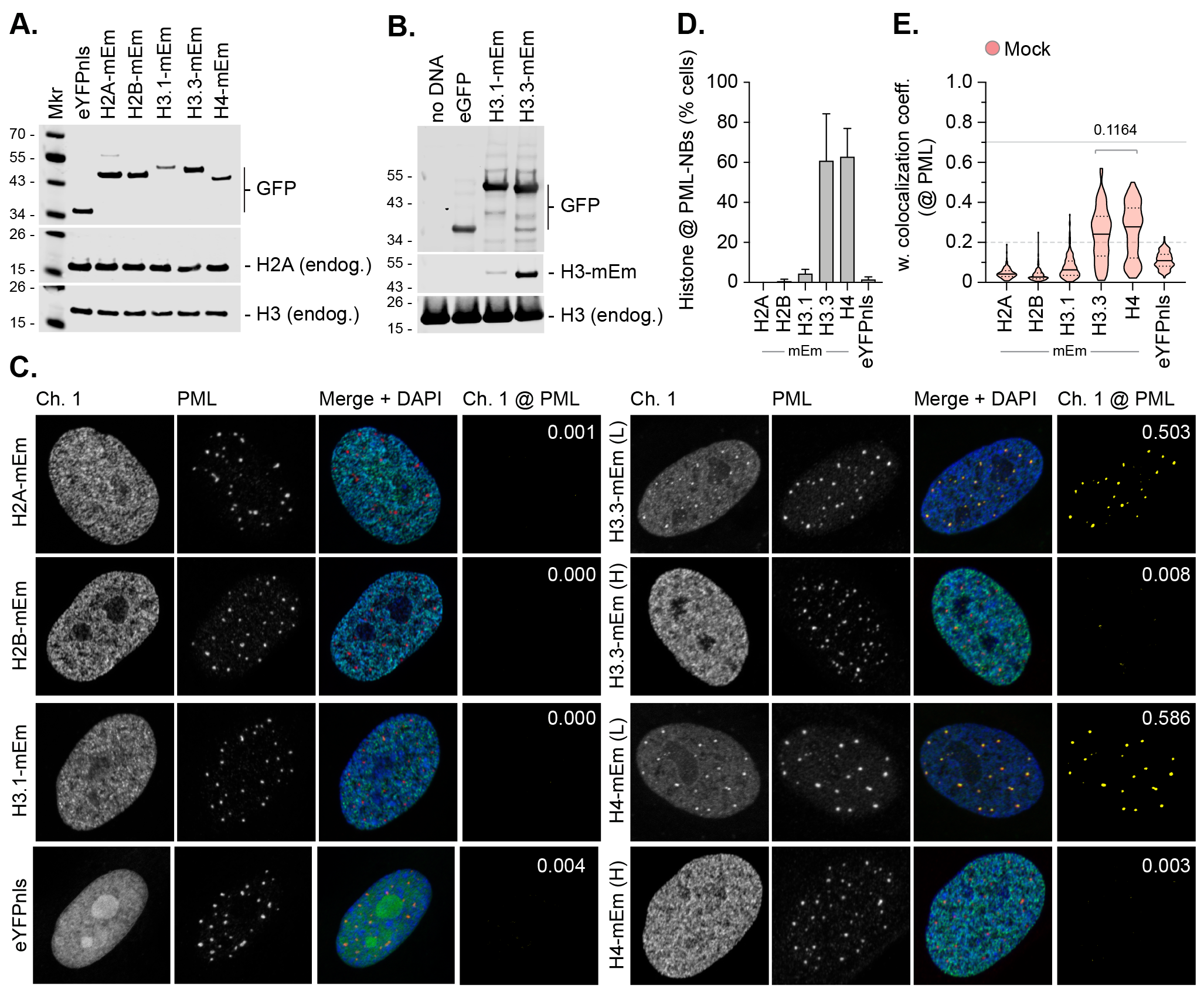

Supplement: S2 Fig — HFt cells were stably transduced with lentiviral vectors encoding C-terminally tagged fluorescent (mEmerald; mEm) histones or eYFPnls (negative control) as indicated. (A) Cells were induced to express proteins of interest for 24 h with doxycycline (DOX) prior to whole cell lysate (WCL) collection and western blotting. Membranes were probed for GFP and endogenous (endog.) histones H2A or H3. (B) RPE cells were transfected with plasmids expressing eGFP, H3.1-mEm, or H3.3-mEm for 24 h prior to WCL collection and western blotting. Membranes were probed for GFP and histone H3. (A/B) Molecular mass markers shown. (C to E) HFt cells were DOX induced for 6 h prior to fixation and indirect immunofluorescence staining for PML (red). Nuclei were stained with DAPI (blue). (C) Confocal microscopy images of histone-mEm or eYFPnls localization at PML-NBs. Cut mask (yellow) highlights regions of colocalization between cellular proteins of interest and PML; weighted (w.) colocalization coefficient (coeff.) shown. Cells expressing high (H) and low (L) levels of H3.3-mEm and H4-mEm are indicated. (D) Quantitation of the percentage of cells that demonstrate histone-mEm or eYFPnls colocalization at PML-NBs. Means and SD shown. (E) Violin plots showing histone-mEm w. colocalization coeff. frequency at PML-NBs: median w. colocalization coeff., solid black line; 25th to 75th percentile range, dotted black lines; coincidence threshold (0.2), dotted grey line; high confidence threshold (0.7), solid grey line. Mann-Whitney U-test, P-value shown. (D/E) N ≥ 150 nuclei per sample condition. (A to E) Data derived from a minimum of three independent experiments. Raw values presented in S1 data. (TIF) [file ppat.1012501.s002.tif]

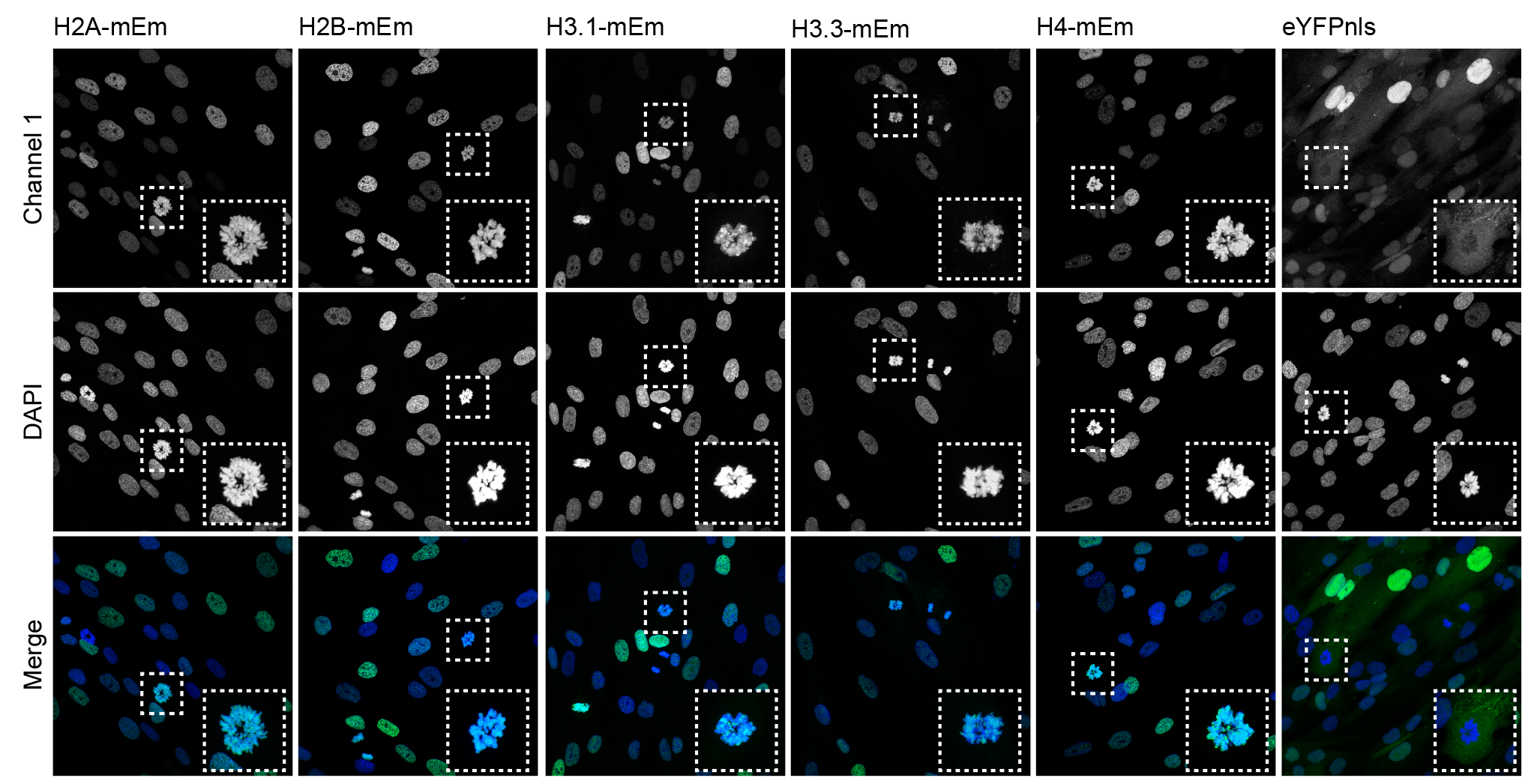

Supplement: S3 Fig — HFt cells stably transduced with lentiviral vectors encoding C-terminally tagged fluorescent (mEmerald; mEm) histones (as indicated) or eYFPnls (negative control) were induced with doxycycline for 6 h prior to fixation. Nuclei were stained with DAPI (blue). Representative x63 objective lens wide-field confocal microscopy images showing histone-mEm localization in mock-treated HFt cells. Dashed boxes show magnified regions of interest highlighting histone-mEm or eYFPnls localization at mitotic chromatin. (TIF) [file ppat.1012501.s003.tif]

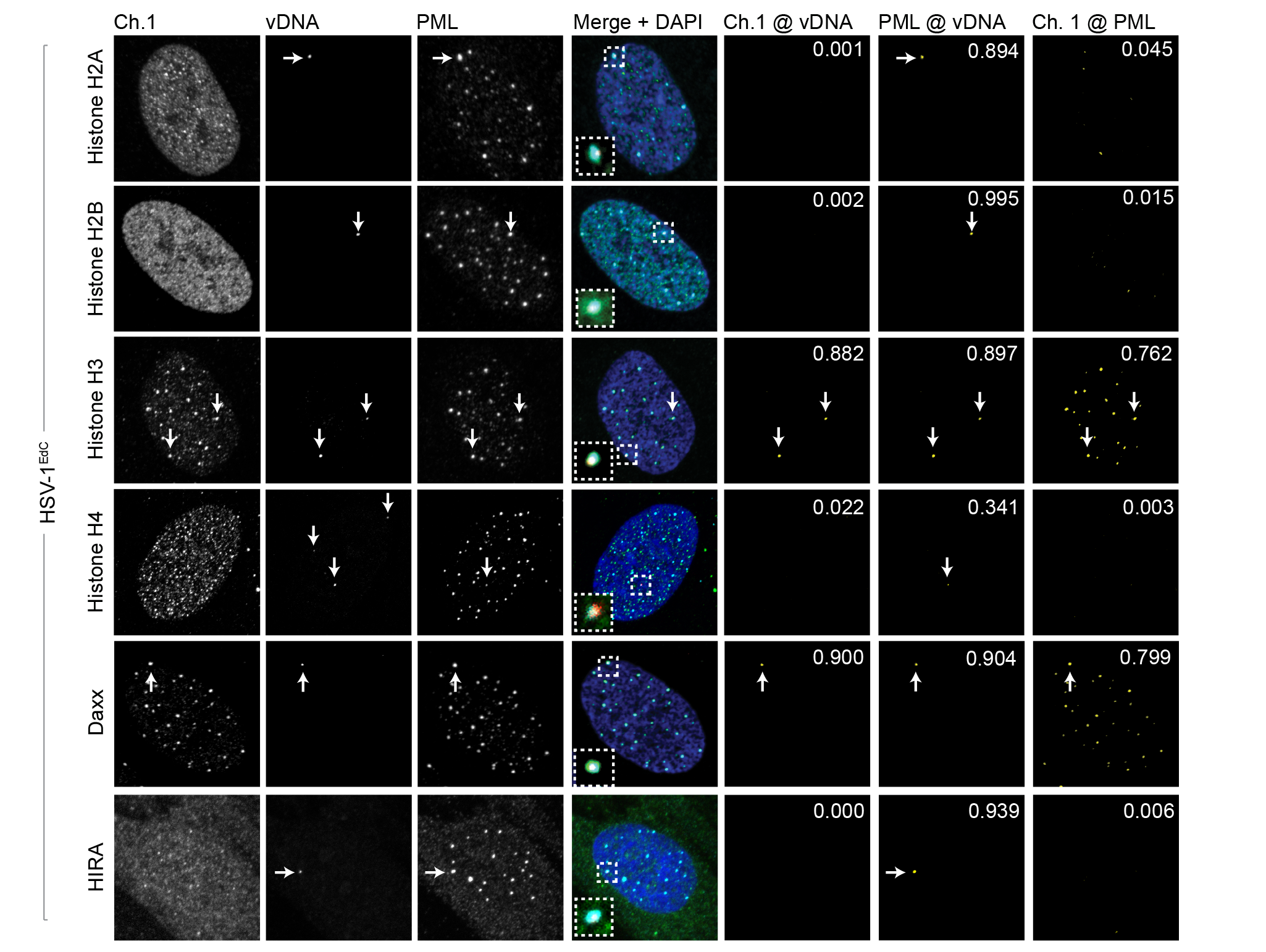

Supplement: S4 Fig — Confocal microscopy images of data presented in Fig 1D to 1G. HFt cells were infected with WT HSV-1EdC (MOI of 1 PFU/cell). Cells were fixed at 90 mpi and stained for Daxx, HIRA, histones H2A, H2B, H3, or H4 (Channel 1 [Ch.1]; green, as indicated) and PML (cyan) by indirect immunofluorescence. vDNA (red) was detected by click chemistry. Nuclei were stained with DAPI (blue). Cut mask (yellow) highlights regions of colocalization between cellular proteins of interest and vDNA or PML (as indicated); weighted colocalization coefficient shown. Dashed boxes show magnified regions of interest. White arrows highlight regions of colocalization at vDNA. (TIF) [file ppat.1012501.s004.tif]

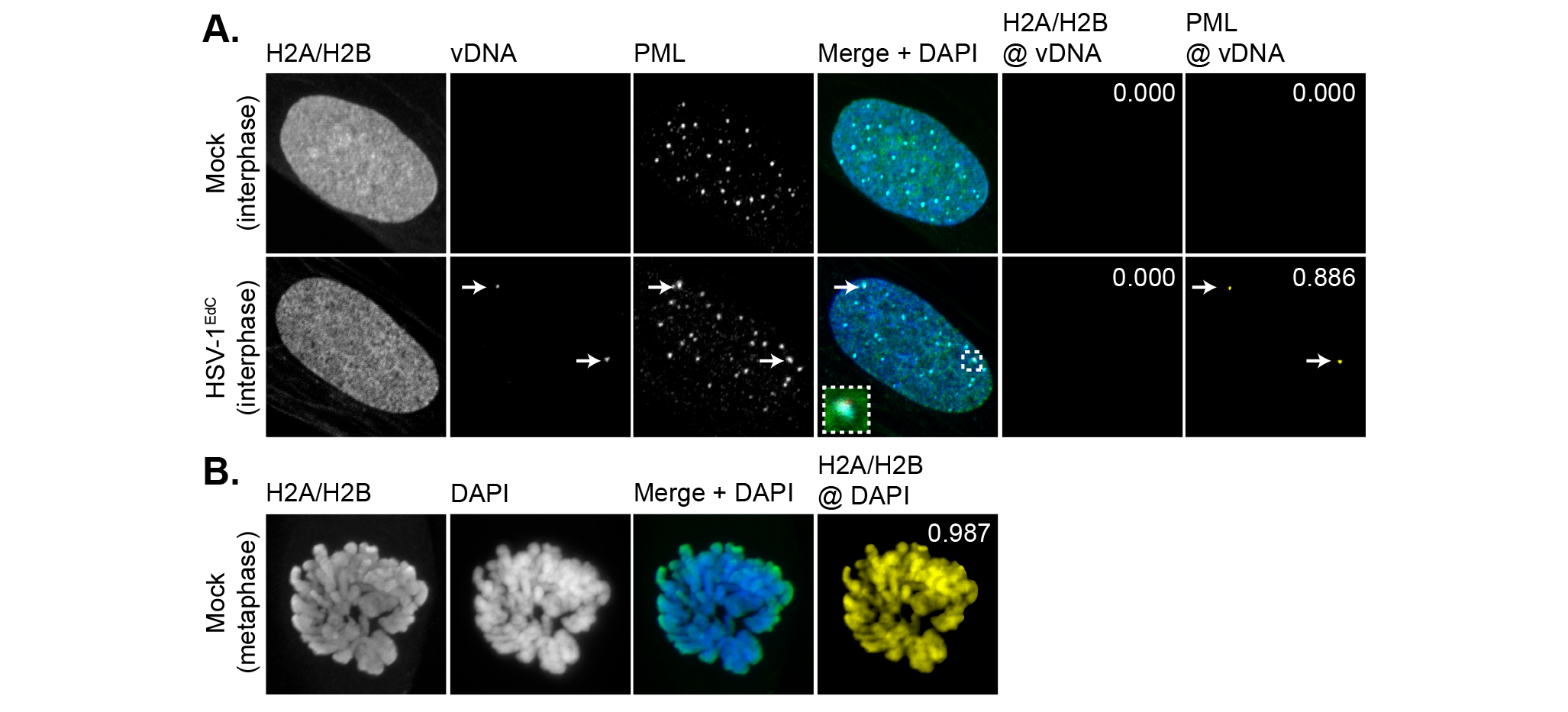

Supplement: S5 Fig — (A/B) Confocal microscopy images of data presented in Fig1E and 1F. HFt cells were mock-treated or infected with WT HSV-1EdC (MOI of 1 PFU/cell). Cells were fixed at 90 mpi and stained for heterodimeric histone H2A/H2B (green) using a fluorescently conjugated nanobody and PML (cyan) by indirect immunofluorescence. vDNA (red) was detected by click chemistry. Nuclei were stained with DAPI (blue). Cut mask (yellow) highlights regions of colocalization between H2A/H2B dimers or PML and vDNA or cellular chromatin; weighted colocalization coefficient shown. Dashed box shows magnified region of interest. White arrows highlight regions of colocalization at vDNA. (B) Localization of histone H2A/H2B heterodimers to mitotic chromatin in mock-treated HFt cells. (TIF) [file ppat.1012501.s005.tif]

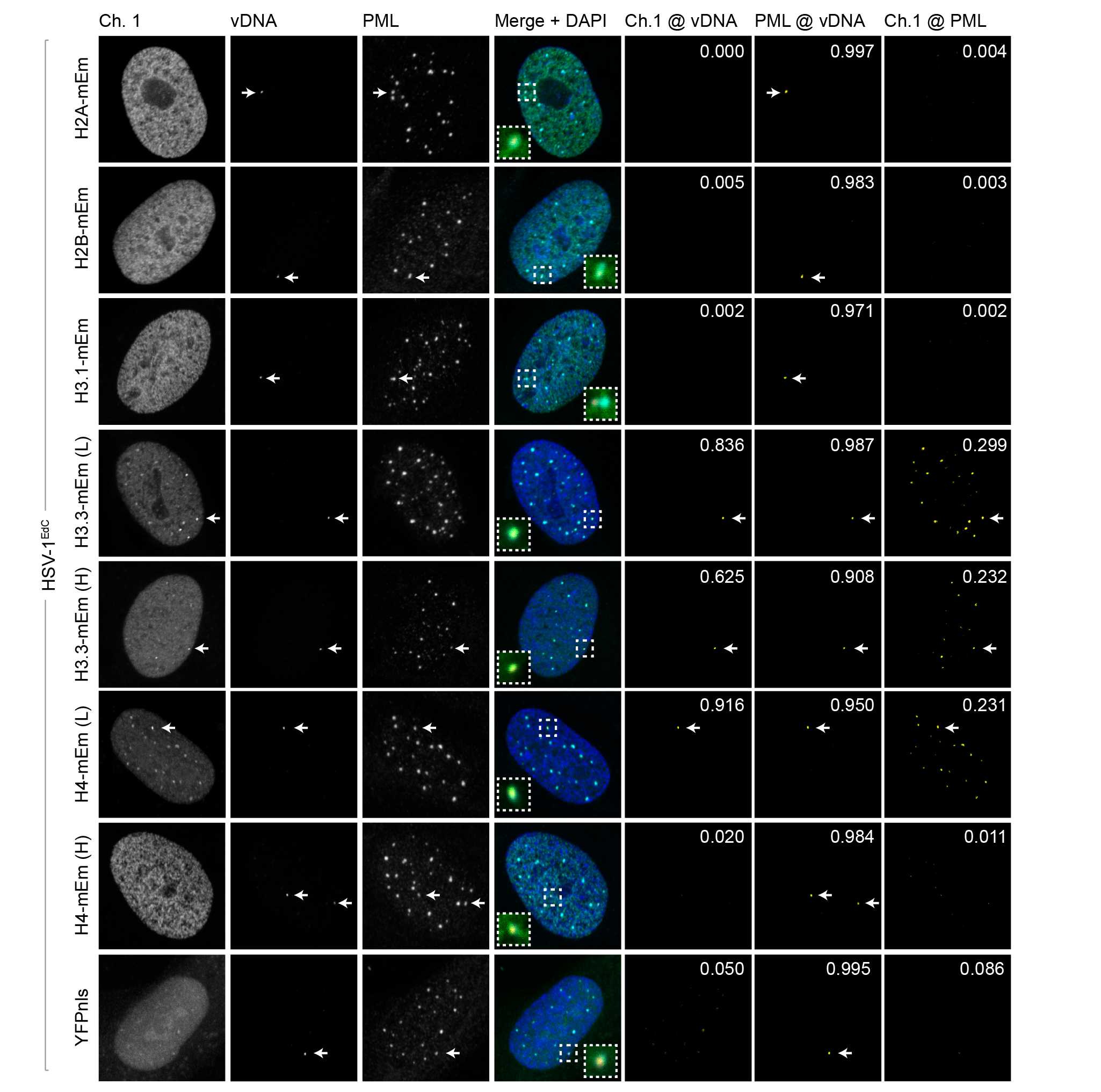

Supplement: S6 Fig — HFt cells stably transduced with lentiviral vectors encoding C-terminally tagged fluorescent (mEmerald; mEm) histones or eYFPnls (negative control) (Channel 1 [Ch.1]; green, as indicated) were induced with doxycycline for 6 h prior to infection with WT HSV-1EdC (MOI of 1 PFU/cell). Cells were fixed at 90 mpi and stained for PML (cyan) by indirect immunofluorescence and vDNA (red) by click chemistry. Nuclei were stained with DAPI (blue). Cut mask (yellow) highlights regions of colocalization between cellular proteins of interest or vDNA (as indicated); weighted colocalization coefficient shown. Dashed boxes show magnified regions of interest. White arrows highlight regions of colocalization at vDNA. Cells expressing high (H) and low (L) levels of H3.3-mEm and H4-mEm are indicated. (TIF) [file ppat.1012501.s006.tif]

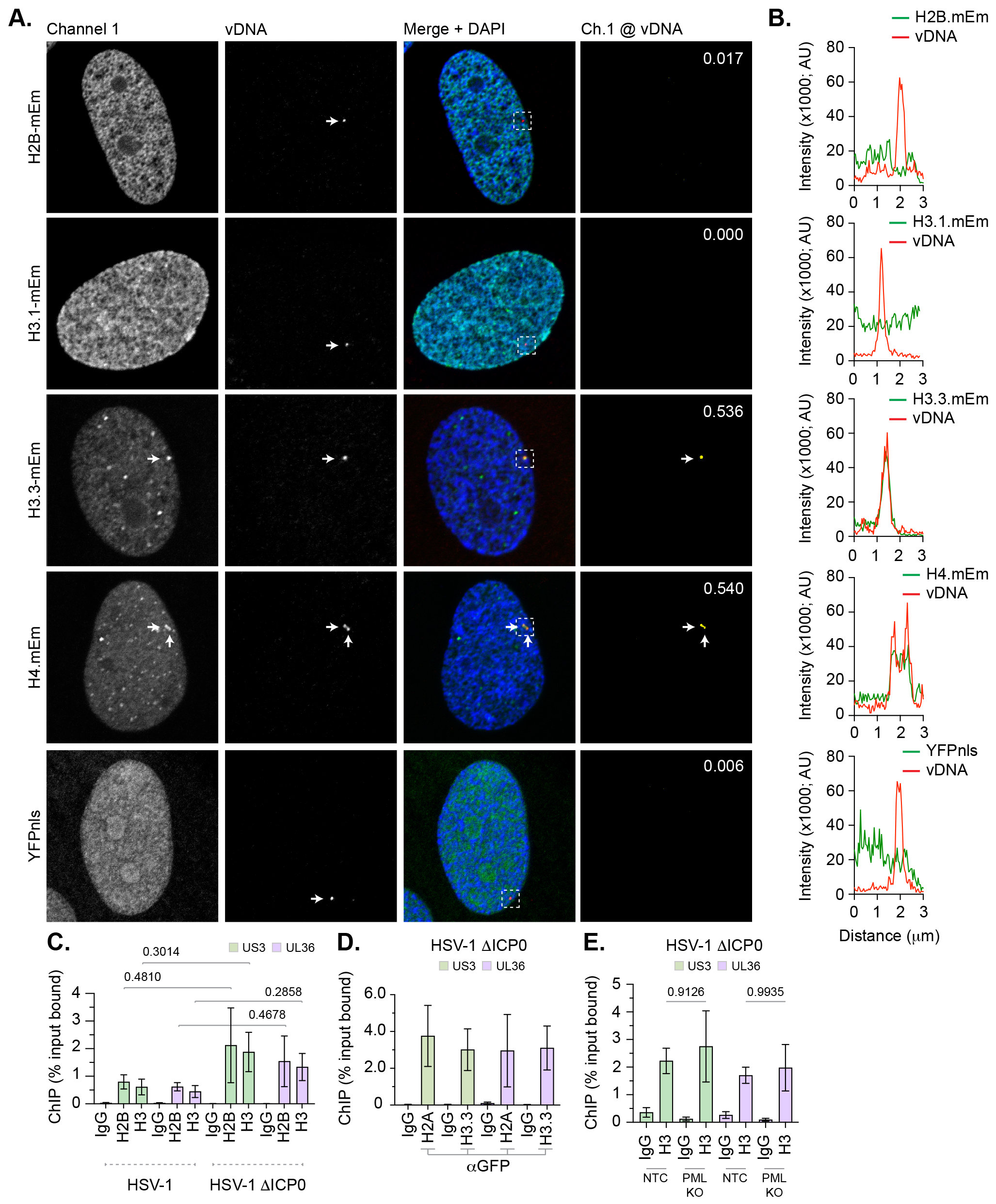

Supplement: S7 Fig — (A) HFt cells stably transduced with lentiviral vectors encoding C-terminally tagged fluorescent (mEmerald; mEm) histones or eYFPnls (negative control) (Channel 1 [Ch.1]; green, as indicated) were induced with doxycycline for 6 h prior to infection with EdC-labelled HSV-1 ICP0-null mutant (ΔICP0; MOI of 1 PFU/cell). Cells were fixed at 90 mpi and vDNA (red) detected by click chemistry. Nuclei were stained with DAPI (blue). Cut mask (yellow) highlights regions of colocalization between mEm-tagged histones and vDNA (as indicated); weighted colocalization coefficient shown. White arrows highlight sub-nuclear localization of vDNA and corresponding regions of histone colocalization. (B) Fluorescent intensity profiles of mEm-tagged histones and vDNA at nuclear regions of interest (dashed boxes in A). (C to E) HFt (C), mEm-tagged histone expressing HFt cells (D), or HFt NTC or PML KO cells (E) were infected with WT or ICP0-null mutant HSV-1 (MOI of 3 PFU/cell; as indicated). Chromatin extracts were prepared at 90 mins post-infection (mpi; post-addition of virus) and subjected to ChIP using ChIP-grade anti-histone (H2A, H2B or H3) and GFP antibodies or species-matched IgG (negative control). Bound viral DNA (vDNA) was quantified by qPCR using probes specific to HSV-1 US3 or UL36. Values were normalized to input loading controls and presented as percentage (%) input bound. Means and SEM shown. Data derived from a minimum of three independent experiments. Raw values presented in S1 Data. (C/E) Paired two-tailed t test, P-values shown. (TIF) [file ppat.1012501.s007.tif]

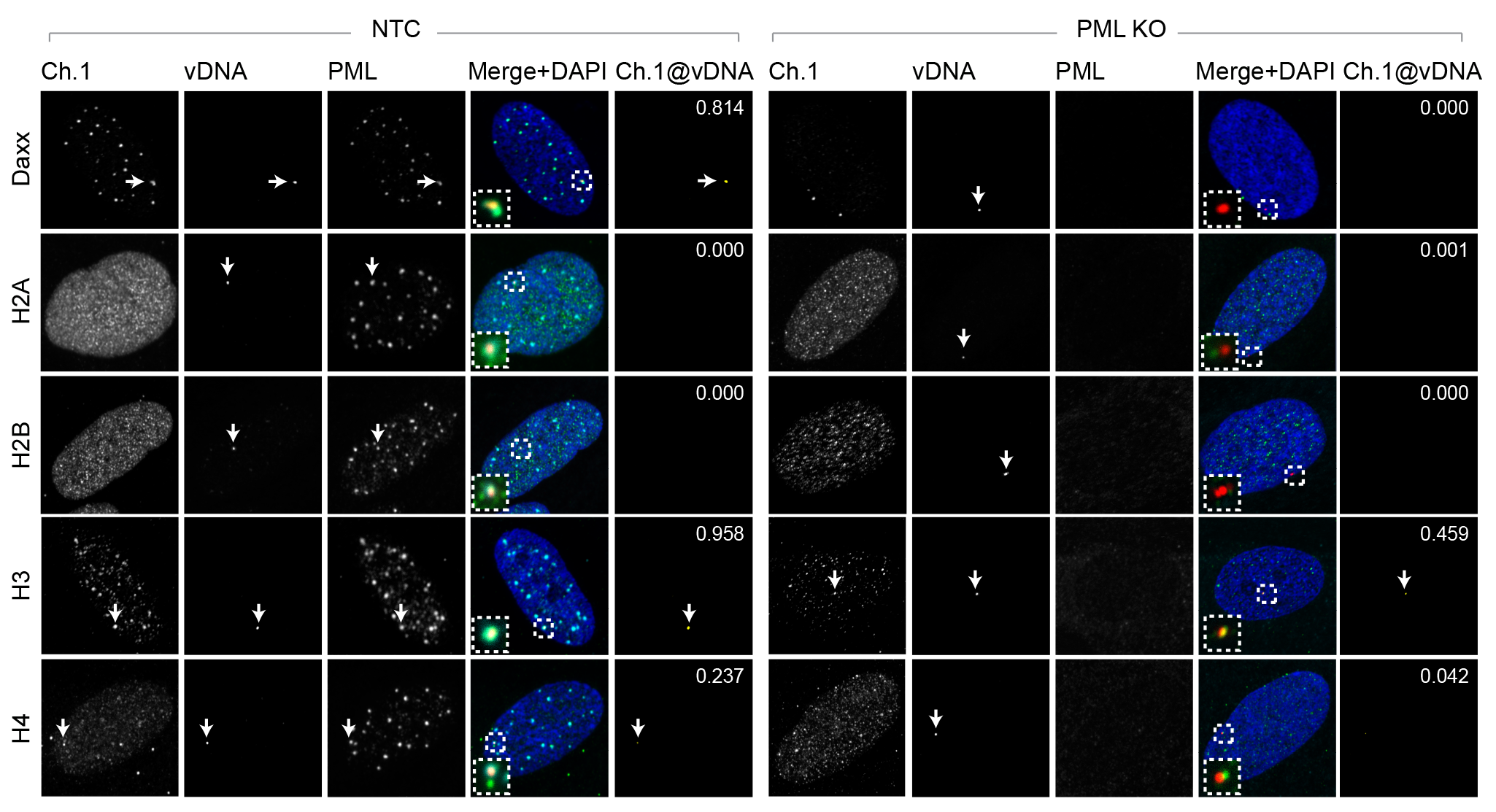

Supplement: S8 Fig — Confocal microscopy images of data presented in Fig 5C and 5D. NTC and PML KO HFt cells were infected with WT HSV-1EdC (MOI of 1 PFU/cell). Cells were fixed at 90 mpi and stained for Daxx, histones H2A, H2B, H3, or H4 (green, as indicated) and PML (cyan) by indirect immunofluorescence. vDNA (red) was detected by click chemistry. Nuclei were stained with DAPI (blue). Cut mask (yellow) highlights regions of colocalization between cellular proteins of interest and vDNA (as indicated); weighted colocalization coefficient shown. White arrows highlight regions of colocalization at vDNA. Dashed boxes show magnified regions of interest. (TIF) [file ppat.1012501.s008.tif]

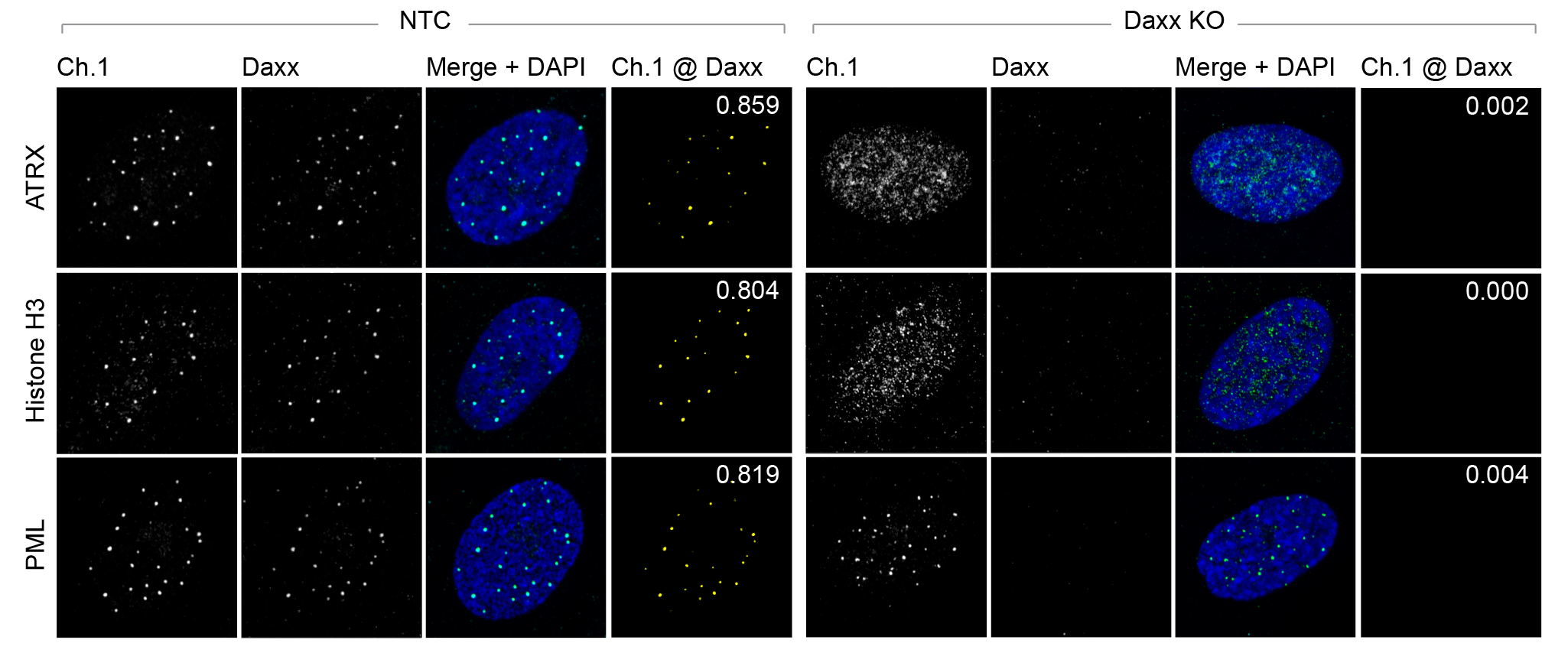

Supplement: S9 Fig — Confocal microscopy images of data presented in Fig 6A. Mock-treated NTC and Daxx KO HFt cells were fixed and stained for PML, ATRX, and histone H3 (green, as indicated) and Daxx (cyan) by indirect immunofluorescence. Nuclei were stained with DAPI (blue). Cut mask (yellow) highlights regions of colocalization between cellular proteins of interest and Daxx (as indicated); weighted (w.) colocalization coefficient (coeff.) shown. (TIF) [file ppat.1012501.s009.tif]

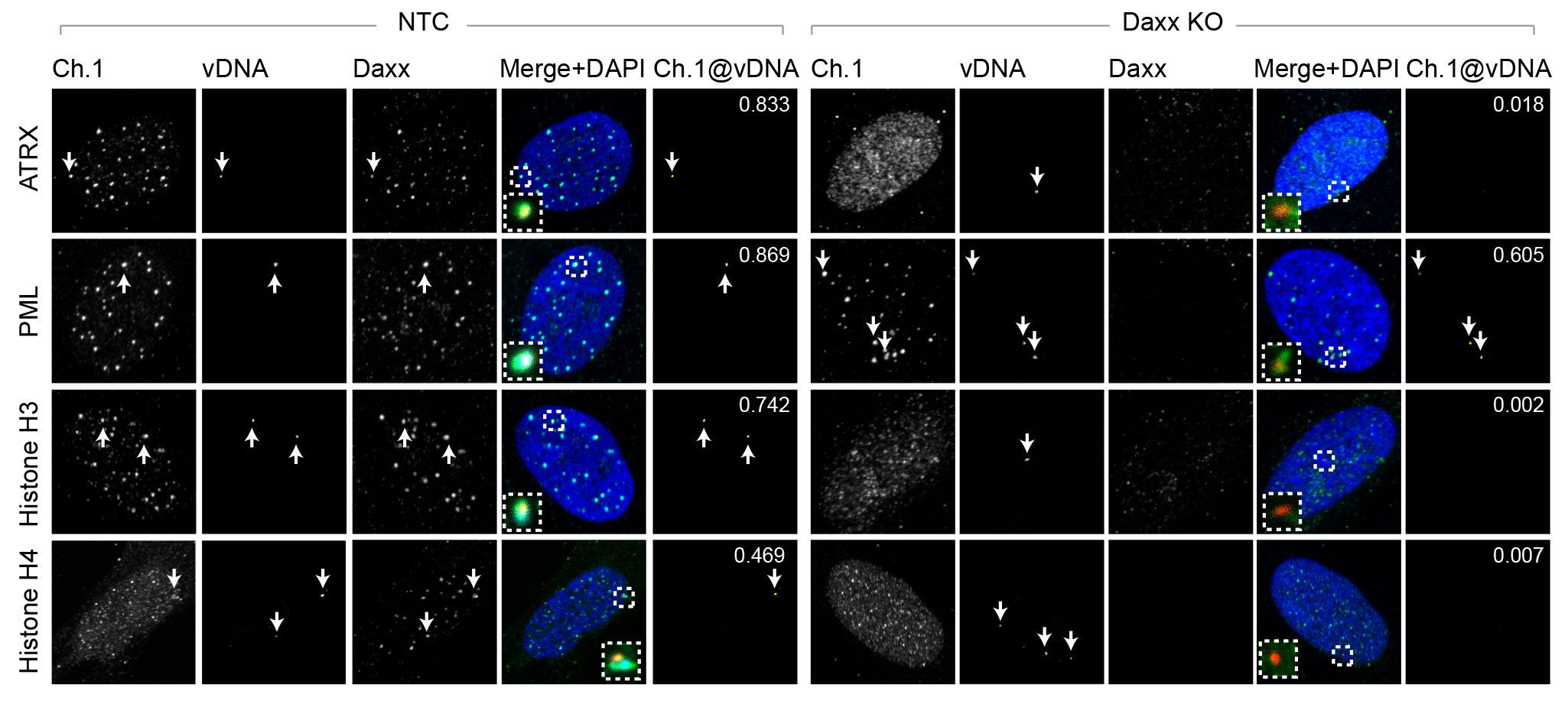

Supplement: S10 Fig — Confocal microscopy images of data presented in Fig 6E and 6F. NTC and Daxx KO HFt cells were infected with WT HSV-1EdC (MOI of 1 PFU/cell). Cells were fixed at 90 mpi and stained for PML, ATRX, histones H3 or H4 (green, as indicated), and Daxx (cyan) by indirect immunofluorescence. vDNA (red) was detected by click chemistry. Nuclei were stained with DAPI (blue). Cut mask (yellow) highlights regions of colocalization between cellular proteins of interest and vDNA (as indicated); weighted colocalization coefficient shown. White arrows highlight regions of colocalization at vDNA. Dashed boxes show magnified regions of interest. (TIF) [file ppat.1012501.s010.tif]

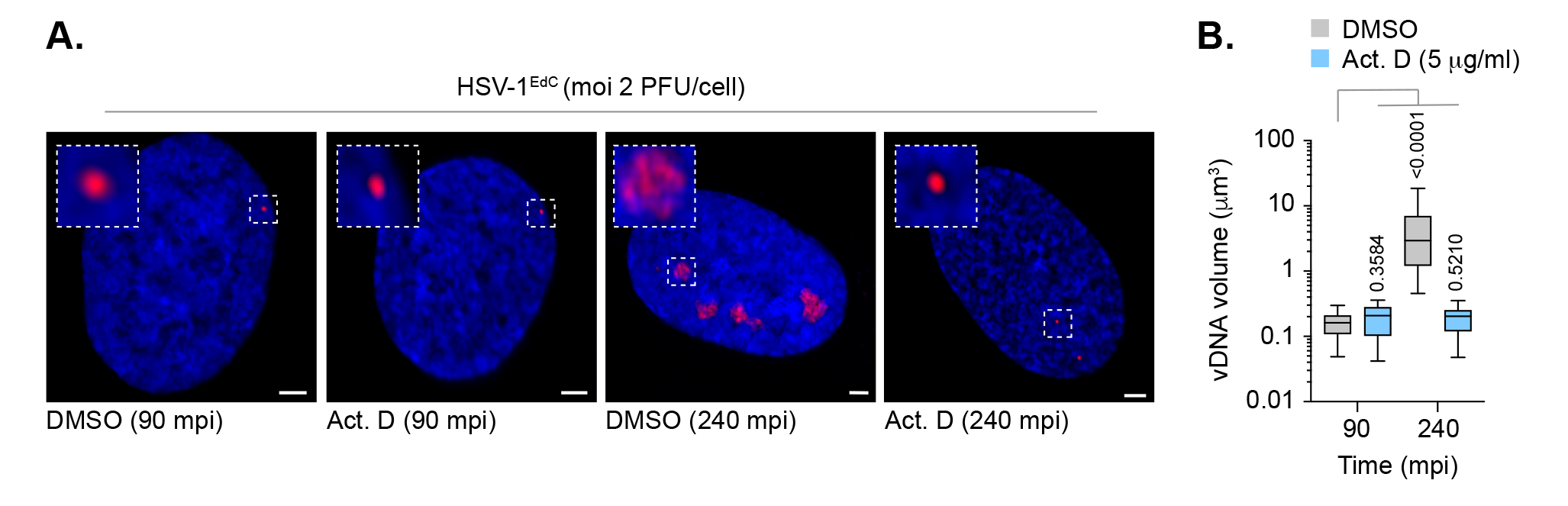

Supplement: S11 Fig — HFt cells were pretreated with DMSO or Actinomycin D (Act. D) for 1 h prior to infection with prelabelled WT HSV-1EdC (MOI of 2 PFU/cell) and overlay 1 h post-absorption with media containing treatment and 2 μM EdC. Cells were fixed at 90 or 240 mpi. vDNA (red) was detected by click chemistry and nuclei were stained with DAPI (blue). (A) Representative high-resolution confocal microscopy images of HSV-1 infected cells over the treatment and time course of analysis (as indicated). Dashed boxes show magnified regions of interest. Scale bars = 2 μm. (B) Quantitation of vDNA foci dimensions (μm3) (as in A). Boxes, 25th to 75th percentile range; whisker, 5th to 95th percentile range; black line, median. N ≥ 95 genome foci per sample condition. Data derived from a minimum of three independent experiments. Kruskal-Wallis one-way ANOVA test, P-values shown. Raw values presented in S1 Data. (TIF) [file ppat.1012501.s011.tif]

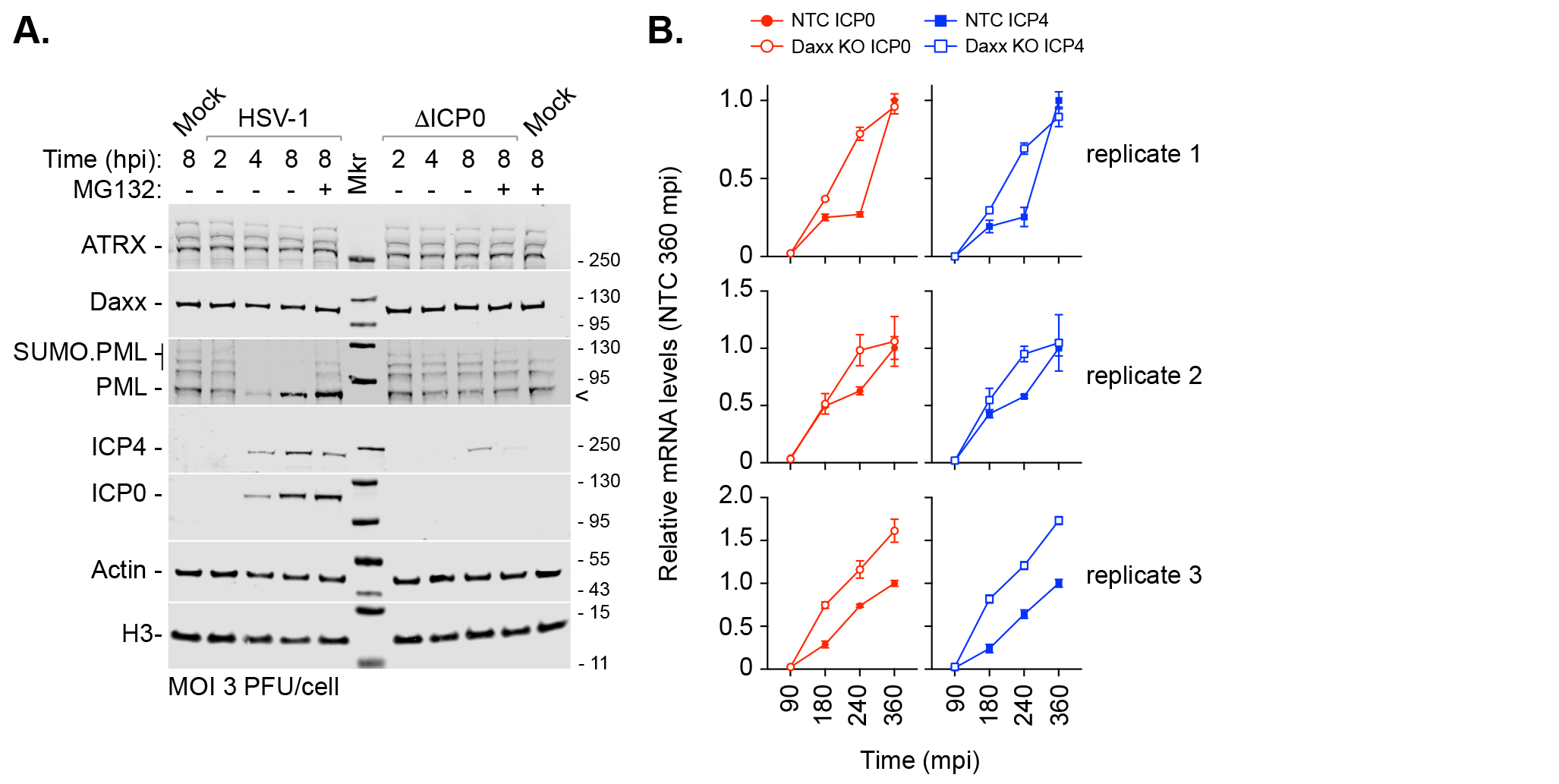

Supplement: S12 Fig — (A) HFt cells were mock-treated or infected with WT or ICP0 null-mutant (ΔICP0) HSV-1 (MOI of 3 PFU/cell) in the absence or presence of the proteasome inhibitor MG132 (5 μM). WCLs were collected at the indicated times (h) post-infection (hpi) and analyzed by western blotting. Membranes were probed for ATRX, Daxx, PML, viral IE proteins (ICP0 and ICP4), histone H3, and actin (loading control). Molecular mass markers shown. < denotes the detection of a non-specific viral protein. (B) Independent replicate experiments of data presented in Fig 8E. NTC and Daxx KO HFt cells were infected with WT HSV-1 (MOI 0.5 PFU/cell). RNA was extracted at the indicated times (minutes post-infection; mpi) and HSV-1 IE transcription (ICP0 and ICP4) quantified by RT-qPCR analysis. Values were normalized to infected NTC cells at 360 mpi. N = 3 independent experiments. Means and SD per experiment shown. Raw values presented in S1 Data. (TIF) [file ppat.1012501.s012.tif]

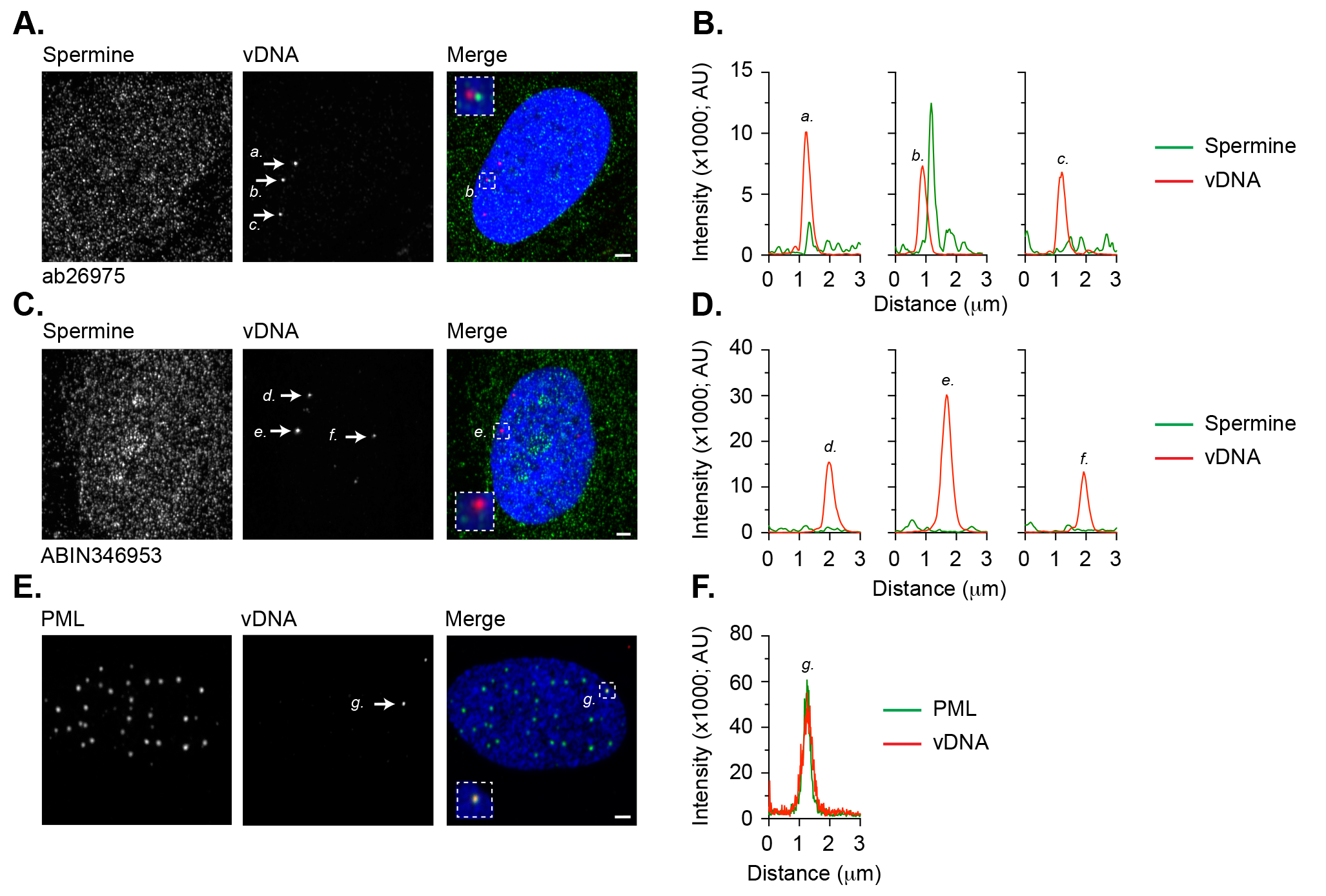

Supplement: S13 Fig — HFt cells were infected with WT HSV-1EdC (MOI of 2 PFU/cell). Samples were fixed at 90 mpi and stained for spermine or PML (green) by indirect immunofluorescence and vDNA (red) by click chemistry. Nuclei were stained with DAPI (blue). (A, C, and E) Representative confocal microscopy images of spermine (A and C) and PML (E) localization with HSV-1 infecting genomes at 90 mpi. Lettered white arrows highlight the detection of nuclear infecting genomes of interest. Dashed boxes show magnified regions of interest (as indicated). (B, D, and F) Emission fluorescence profiles (Arbitrary Units, AU) of spermine and PML localized at vDNA from annotated genomes of interest (as highlighted in A, C, and E, respectively). Raw values presented in S1 Data. (TIF) [file ppat.1012501.s013.tif]
